# Geographic containment and virulence‐resistance trade‐offs drive the evolution of hypervirulent *Klebsiella pneumoniae*

**DOI:** 10.1002/imt2.70077

**Published:** 2025-09-04

**Authors:** Yuchen Wu, Fan Pu, Zelin Yan, Yanyan Zhang, Kaichao Chen, Shengkai Li, Yuezhuo Wang, Heyuan Lun, Tingting Qu, Jing Wang, Heng Li, Danxia Gu, Sheng Chen, Ping He, Rong Zhang, Zhemin Zhou

**Affiliations:** ^1^ Department of Clinical Laboratory Second Affiliated Hospital of Zhejiang University, School of Medicine Hangzhou China; ^2^ The Second Affiliated Hospital of Soochow University, Cancer Institute, Suzhou Medical College, Soochow University Suzhou China; ^3^ National Center of Technology Innovation for Biopharmaceuticals, Suzhou Biomedical Industry Innovation Center Suzhou China; ^4^ State Key Lab of Chemical Biology and Drug Discovery and The Department of Food Science and Nutrition The Hong Kong Polytechnic University Kowloon Hong Kong China; ^5^ Shenzhen Key Lab for Food Biological Safety Control The Hong Kong Polytechnic University Shenzhen Research Institute Shenzhen China; ^6^ Department of Immunology and Microbiology Shanghai Jiao Tong University School of Medicine Shanghai China; ^7^ National Key Laboratory of Intelligent Tracking and Forecasting for Infectious Diseases, National Institute for Communicable Disease Control and Prevention, Chinese Center for Disease Control and Prevention Beijing China

**Keywords:** carbapenemase‐encoding plasmids, clonal complex 23, hypervirulent carbapenem‐resistant *Klebsiella pneumoniae*, population dynamics

## Abstract

The emergence of hypervirulent carbapenem‐resistant *Klebsiella pneumoniae* (hvCRKP) represents an alarming convergence of enhanced virulence and extensive drug resistance. Here, we present a comprehensive genomic analysis of 2563 clonal complex 23 (CC23) isolates from 62 countries spanning 1932–2024. Our findings reveal that CC23‐K1, the dominant hypervirulent sublineage, emerged approximately 170 years ago and diversified into seven major clades with distinct regional dominance. We observe that carbapenem resistance in CC23‐K1 exhibits notable instability, with at least 130 independent acquisitions and 20 losses of resistance genes, suggesting an evolutionary trade‐off between hypervirulence and antimicrobial resistance. Experimental validation demonstrates that capsule production physically impedes plasmid conjugation, while isolates carrying *bla*_KPC‐2_, *bla*_NDM‐1_, or *bla*_NDM‐5_ frequently exhibit substantial deletion of virulence determinants. Conversely, *bla*_OXA‐48_‐carrying isolates maintain virulence gene integrity, potentially due to their lower hydrolytic activity and reduced fitness costs. The geographic distribution of these resistance mechanisms correlates with regional antimicrobial usage patterns, with European countries with moderate carbapenem use favoring *bla*_OXA‐48_ in CC23, while Asian countries with higher consumption show patterns favoring high‐efficiency carbapenemases incompatible with complete virulence determinants. We also identified core genomic regions with significantly higher mutation rates in resistant isolates, particularly affecting pathways involved in oxidative phosphorylation and reactive oxygen species production. These findings provide additional insights into CC23 evolution and geographical spread, complementing existing knowledge of carbapenemase distribution patterns observed across *K. pneumoniae* lineages.

## Highlights

- CC23‐K1 hypervirulent *K. pneumoniae* shows strong geographic compartmentalization with distance‐driven transmission patterns globally.
- Carbapenem resistance exhibits remarkable instability with >130 acquisitions and frequent losses.
- High‐efficiency carbapenemases (*bla*_KPC_/*bla*_NDM_), prevalent in Asia, are incompatible with hypervirulence, while low‐efficiency *bla*_OXA‐48_ in Europe preserves virulence determinants.

## Graphical abstract

The global emergence of hypervirulent, carbapenem‐resistant *Klebsiella pneumoniae* (hvCRKP) poses a paradox: why do such dangerous clones remain geographically confined? By analyzing over 2500 CC23 genomes across 90 years, we uncover a core evolutionary constraint—virulence and resistance rarely coexist without trade‐offs. Potent carbapenemases (*bla*_KPC_, *bla*_NDM_) are frequently linked to large‐scale deletions in virulence loci, while capsule production physically hinders plasmid uptake. In contrast, low‐activity enzymes like *bla*_OXA‐48_ preserve full virulence. Experimental validation and mutational profiling reveal how metabolic adaptation and structural barriers limit convergence. Our findings reveal an evolutionary balancing act that restrains global dissemination of hvCRKP—and suggest new levers for containment and control.

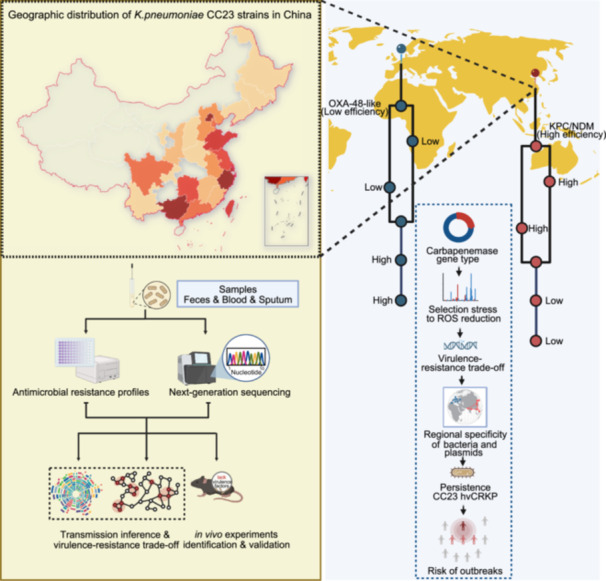

## INTRODUCTION

*Klebsiella pneumoniae* (*K. pneumoniae*) is a major cause of both healthcare‐ and community‐associated infections, ranging from pneumonia to life‐threatening bloodstream infections [1]. This bacterium exhibits significant genetic diversity, with multi‐drug‐resistant (MDR‐KP) strains linked to hospital‐acquired infections, and hypervirulent strains (hvKP) traditionally linked to community‐acquired infections with lower resistance profiles [2, 3]. However, the emergence of hypervirulent carbapenem‐resistant *K. pneumoniae* (hvCRKP), which combines both virulence and antimicrobial resistance, has raised significant concerns [4]. Among these, clonal complex (CC) 23 strains, encompassing sequence type (ST) 23 and its single‐locus variants, have emerged in Europe as a key focus, raising questions about potential changes in the epidemiology of these infections, though their clinical significance compared to other carbapenem‐resistant *K. pneumoniae* lineages remains to be firmly established [4, 5].

CC23 strains, especially those with the K1 capsular serotype, have long been recognized as hypervirulent due to their possession of key virulence determinants, including the regulator of mucoid phenotype gene (*rmpA*/*rmpA2*), three iron acquisition systems (*iucABCD*‐*iutA*, *iroBCDN*, and *Yersinia* high‐pathogenicity island), and the colibactin genotoxin (*clbA‐S*) [6, 7]. More recently, these traditionally hypervirulent strains have been increasingly associated with multi‐drug resistance, including carbapenem resistance, particularly in Europe [8]. Several clusters of CC23 hvCRKP isolates have been linked to persistent transmission in healthcare facilities in Ireland, Latvia, and France, suggesting a troubling trend of localized outbreaks and potential within‐country transmission [4]. Understanding the interplay between virulence and resistance in these strains may provide insights applicable to other emerging high‐risk clones.

While previous studies have characterized the population structure and evolutionary history of CC23 strains, questions remain about the specific mechanisms governing the acquisition and maintenance of carbapenem resistance in hypervirulent backgrounds [9, 10]. While some reports suggest that the acquisition of carbapenem resistance in hvKP strains does not significantly compromise their virulence, others point to potential incompatibilities between these traits [10, 11]. The coexistence of hypervirulence and antimicrobial resistance in a single strain presents an evolutionary challenge, and understanding the genetic and phenotypic interactions between these traits could inform surveillance and control strategies.

In this study, we present a comprehensive analysis of an international collection of *K. pneumoniae* ST23 isolates, encompassing samples from 50 countries and spanning 90 years. By integrating genomic epidemiology with experimental validation, we explore the population structure, evolutionary dynamics, and geographic distribution of CC23‐K1. We investigate patterns of carbapenem resistance acquisition and examine potential trade‐offs between hypervirulence and specific carbapenemase types, which applies to all hvCRKPs independent of their genetic background.

## RESULTS

### Global population structure and geographic distribution of *K. pneumoniae* CC23

**Figure 1:**
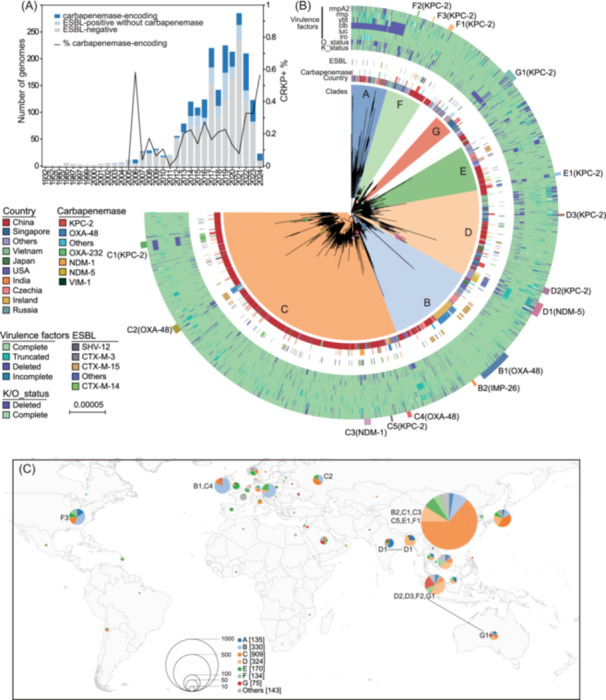
Global distribution and evolutionary dynamics of *Klebsiella pneumoniae* (*K. pneumoniae*) clonal complex 23 (CC23) K1 lineage. (A) Temporal distribution of *K. pneumoniae* CC23 isolates from 1932 to 2024, showing the number of genomes (bars) stratified by their predicted antimicrobial resistance profiles. The black line represents the percentage of carbapenemase‐encoding strains among all CC23 over time. ESBL: extended‐spectrum β‐lactamase. (B) Maximum‐likelihood phylogenetic tree of the CC23‐K1 lineage (*n* = 2077) with recombination regions removed, revealing seven distinct clades (Clades A–G) shown in different colors. The outer rings display the distribution of key metadata, including virulence factors, antimicrobial resistance determinants (ESBL and carbapenemase genes), country of isolation, and clade assignment. Labeled branches (B1–G1) indicate major carbapenem‐resistant *Klebsiella pneumoniae* (CRKP) clusters defined by carbapenemase type (in parentheses). K/O_status: whether the genes associated with capsule (K) or O‐antigen synthesis were complete or partially/completely deleted. (C) Geographic distribution of CC23‐K1 clades across the globe. Pie charts represent the relative abundance of different clades in each geographic region, with size proportional to the number of isolates as indicated in the legend.

Our longitudinal surveillance of *K. pneumoniae* in a teaching hospital in Hangzhou, Zhejiang, revealed that CC23 accounted for 9.5% of carbapenem‐susceptible isolates and <1% of carbapenem‐resistant isolates over the past 30 years (Figure S1), except for a notable peak in CC23 CRKP isolation occurred between 2006 and 2008, reaching 15%, 3 years before the earliest reports from other regions [12]. None of the recorded CC23 infections resulted in fatality. We sequenced 151 CC23 isolates from six provinces in China (1999–2024) and integrated them with publicly available data to form a comprehensive data set of 2563 isolates from 62 countries, spanning from 1932 to 2024 (Figure 1A).

Phylogenetic analysis indicated that CC23 is polyphyletic, comprising at least 40 STs (Table S1) across 22 distinct branches (Figure S2 and Table S2). Notably, several sequence types (STs), including ST23, appeared in multiple branches, likely due to frequent recombination. Core genome‐based designation using cgLINcode further confirmed the presence of at least 13 different sublineages and 27 genomic clonal groups (Figure S2 and Table S2) [13]. Most isolates (2220/2563, 86.6%) clustered into a single branch (CC23‐K1), named after their K1 capsular type, a marker associated with hypervirulence. However, cgLINcode, and other cgMLST‐based approaches lack the allelic resolution needed to distinguish deeper, century‐scale sublineages within CC23‐K1, a limitation inherent to distance‐based allele schemes. In contrast, phylogeny‐aware, whole‐genome single‐nucleotide polymorphisms (SNP) approaches capture the fine‐scale mutational signal required to resolve these historical branching events.

Analysis of the CC23‐K1 lineage, after the removing recombination regions, identified seven major clades (A–G) in 93.6% of isolates (2077/2220) (Figure 1B, Figure S3A) with distinct geographic distributions: Clade C predominated in East Asia, Clade D in Southeast Asia, and Clades B and E in Europe (Fisher's exact test, all *p* < 1e‐8; Figure 1C). We identified 339 carbapenemase‐producing isolates within CC23‐K1, primarily carrying *bla*_KPC‐2_ (129 isolates, 38%), *bla*_OXA‐48_ (94, 28%), *bla*_NDM‐1_ (37, 11%), and *bla*_NDM‐5_ (29, 9%) (Table S3). These carbapenemase‐producing isolates accounted for approximately 20% of CC23‐K1 isolates since 2013 (Figure 1A). Carbapenemase distribution showed marked regional specificity: *bla*_KPC‐2_ predominated in China, Singapore, and the US, while *bla*_OXA‐48_ and *bla*_NDM‐5_ were most common in Europe and Bangladesh, respectively (Figure S3B).

### Repetitive acquisitions and losses of carbapenem resistance

The CC23‐K1 lineage acquired carbapenemase genes independently at least 130 times, forming distinct clusters across the phylogeny (Figure 1B). Only 15 clusters contained three or more CRKP isolates, which we designated as CRKP clusters B1‐G1. A pairwise comparison of the CRKPs revealed that 94% of strains within the same cluster differed by fewer than 93 SNPs (Figure S4A), underscoring their recent emergence.

Network analysis of all CC23‐K1 strains using the same SNP threshold yielded 254 distinct modules (Figure S4B). Notably, 36 (95%) of the 38 CRKP‐associated large modules (≥3 isolates) also included carbapenem‐susceptible *K. pneumoniae* (CSKP) (Figure S4C). The intermingling between CRKPs and CSKPs indicates frequent cycles of gene acquisition and loss, likely driven by bidirectional selective pressure.

### Population dynamics and distance‐driven transmissions

**Figure 2:**
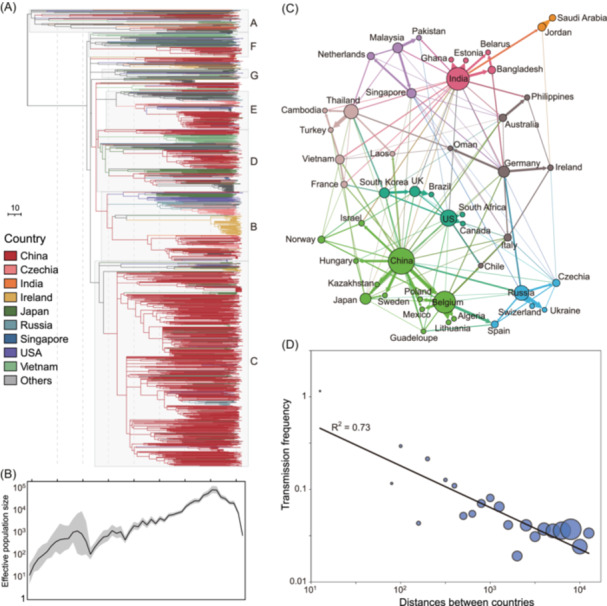
Evolutionary dynamics and geographic transmission patterns of *K. pneumoniae* CC23‐K1 lineage. (A) Time‐calibrated phylogenetic tree of the CC23‐K1 lineage constructed using TreeTime, with branches colored by country of origin according to the legend. The seven major clades (Clades A–G) are indicated on the right of the tree. Note that (a) shares the same temporal axis (years) as in part b, to facilitate direct comparison of phylogenetic divergence and demographic expansion. (B) Effective population size dynamics of CC23‐K1 over time, shown on a logarithmic scale. The black line represents the median estimate, with the gray shaded area indicating the 95% confidence interval. (C) International transmission network of CC23‐K1 lineage. Nodes represent countries, with node size proportional to the number of isolates. Edges connecting nodes indicate transmissions and are sized proportional to the relative frequencies of the source country contributing to the targets. Countries are clustered into distinct modules represented by different colors, revealing the geographic specificity of transmission patterns. The modules are estimated using Louvain algorithm. (D) Correlation between transmission frequencies and geographic distances between countries on a log‐log scale. The country distances were estimated as the minimum distance between major cities in two countries, retrieved from https://www.geonames.org. The country pairs were binned by their log‐transformed distances for every 0.1, with circles sized proportional to the number of associated pairs. A significant negative correlation is observed (*R* ^2^ = 0.73), demonstrating that transmission frequency decreases as geographic distance increases.

TreeTime analysis of the CC23‐K1 lineage estimated its origin around 1857 (95% CI: 1843–1875) (Figure 2A) with a significant temporal signal validated by randomization tests (Figure S5A–C). Independent BEAST2 runs with four random subsets of the CC23‐K1 strains similarly estimated the origin of CC23‐K1 to be between 1834 and 1888 (Figure S5D–H). All evolutionary analyses indicated a major expansion of effective population size in the 1940s, coinciding with accelerated international transmission (Figure 2B).

Direct ancestral state reconstruction suggested that China was the primary source of international transmission, contributing to 55% (92/167) of transmissions (Figure S6A). To account for geographic overrepresentation in Europe and East Asia, we implemented a down‐sampling strategy, randomly selecting up to 10 genomes per country for geographic state reconstruction (see Methods) [14]. Phylogeographic reconstruction using these balanced datasets (100 replicate subsamples) confirmed the overall transmission trends, with China remaining the dominant transmission source (22.2% of transmissions), followed by Belgium (18.5%) and India (12.2%) (Figure S6B–F). Network analysis revealed eight distinct country modules with strong geographic specificity (Figure 2C). We observed a significant negative correlation between transmission frequency and geographic distance between countries (*R* ^2^ = 0.73) (Figure 2D and Table S4), with countries <500 km apart experiencing five‐fold more transmissions than those >3000 km apart (0.16 vs. 0.03), highlighting the role of geographic barriers in CC23‐K1's dissemination.

### Geographic specificity of the carbapenemase‐carrying plasmids

We identified 28 distinct carbapenem resistance plasmids in CC23‐K1 isolates, each strongly associated with specific carbapenemase genes, according to the plasmid typing (PT) scheme in Li et al. [2]. The most prevalent were PT_804 (93 isolates, *bla*_OXA‐48_), PT_360 (45, *bla*_KPC‐2_), PT_3142 (36, *bla*_KPC‐2_), and PT_695 (26, *bla*_NDM‐5_).

**Figure 3:**
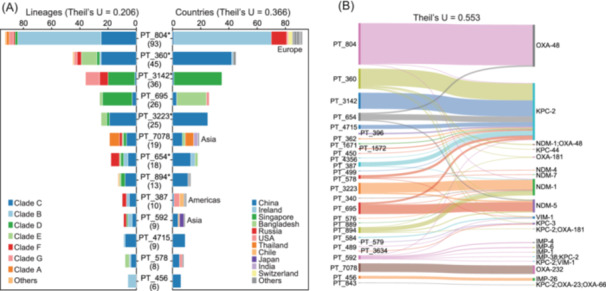
Geographic distribution and genetic associations of carbapenemase‐carrying plasmids in *K. pneumoniae* CC23‐K1. (A) Distribution of carbapenemase‐carrying plasmids among different bacterial lineages (left) and countries (right). The x‐axis represents the number of isolates. Each plasmid type (PT) is labeled with the total number of isolates in parentheses. Colors in the left panel represent different CC23‐K1 clades, while colors in the right panel represent different countries. Major continental associations are annotated (Europe, Asia, Americas), showing strong regional specificity of certain plasmids. (B) Sankey diagram illustrating the relationships between plasmid types (left) and carbapenemase genes (right). The width of each flow corresponds to the frequency of association between a specific plasmid type and carbapenemase gene.

Theil's U tests indicated that plasmid distribution was only weakly associated with host bacterial clades (U = 0.206) (Figure 3A), suggesting frequent horizontal transfer, even between different clonal complexes or species (Table S5). For example, PT_3142 was detected in 88 distinct sequence types and 11 other Enterobacteriaceae species, including *Salmonella enterica* and *Shigella flexneri*.

In contrast, geographic origin explained 50% more variation in plasmid distribution than bacterial clades (Theil's U = 0.366; Figure 3A), with eight plasmids predominantly (≥80% of isolates) detected in single countries and four others showing continent‐specificity. Phylogenetic analysis reinforced these geographic patterns: PT_3142 plasmids from Singapore, hosted by strains from five different clades, showed indistinguishable core genome sequences (Figure S7A). Similarly, PT_804 plasmids from various European countries formed a tight phylogenetic cluster distinct from Chinese isolates (Figure S7B). These geographic patterns, coupled with the strong association between carbapenemase genes and plasmids (Figure 3B), partially explain the regional distribution of carbapenemase genes and highlight the role of plasmids in maintaining antimicrobial resistance.

### Trade‐off between virulence and antimicrobial resistance

**Figure 4:**
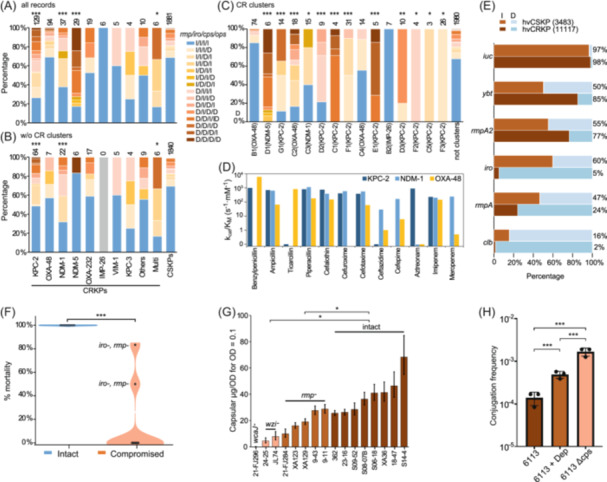
Trade‐off between virulence and antimicrobial resistance in *K. pneumoniae*. (A) Distribution of virulence gene integrity patterns (*rmpA*/*iro*/*cps*/*ops*) among different carbapenemase‐producing *K. pneumoniae* isolates. The *x*‐axis shows carbapenemase types; the *y*‐axis shows the percentage of isolates with each integrity pattern (I = intact, D = deleted/disrupted). The right‐most histogram shows carbapenem‐susceptible *Klebsiella pneumoniae* (CSKP) isolates, for comparison with the CRKPs in the Fisher's exact tests. The numbers of associated isolates for each carbapenemase types were shown above the histograms. Asterisks indicate statistical significance: *p < 0.05, ***p < 0.001. Multi: isolates with multiple carbapenemase genes. (B) Replicates of part a, except that all isolates in CRKP clusters were removed to migrate potential sampling bias. (C) Virulence gene integrity patterns across different CRKP clusters. The right‐most histogram shows all isolates outside of these clusters, including both CSKPs and CRKP singletons. The numbers of associated isolates for each carbapenemase types were shown above the histograms. Asterisks indicate statistical significance: *p < 0.05, **p < 0.01, ***p < 0.001. (D) Hydrolytic efficiency (*k*_cat_/*K*_M_ values) of different carbapenemase enzymes against β‐lactam antibiotics, presented on a logarithmic scale. The data was obtained from http://www.bldb.eu/F-BLDB.php. (E) Prevalence of virulence genes in hypervirulent *K. pneumoniae* (hvKp) isolates (*n* = 14,600) from non‐CC23 lineages, comparing hvCSKP (3483 isolates) and hvCRKP (11,117 isolates). The hvKp isolates were defined based on their virulence scores of ≥3 by Kleborate, which indicates the presence of *iuc* loci. However, some of these iuc loci were still assigned as deleted, due to the disruption of their coding sequences due to mutations or short insertion/deletions. (F) in vivo virulence comparison between strains with intact and mutated virulence genes in a murine infection model. Strains with disrupted virulence factors showed dramatically reduced lethality compared to fully virulent strains (100% mortality) under identical infection conditions (***p < 0.001). (G) Reduced capsule production for isolates with deleted *cps*, *wzi*, and *rmp* genes. Strains with deleted *wzi* or *rmp* genes exhibited reduced capsular thickness, and those with deleted *cps* genes produced no capsules (*p < 0.05). (H) Conjugation frequency in KL1 type *K. pneumoniae* strain with or without capsule. The conjugation frequency of wild type (WT) strain 6113, WT strain 6113 treated with K1 capsular polysaccharide depolymerase (6113 + Dep), and the *wcaJ* gene knockout 6113 strain (6113 ΔCPS) is shown. The donor strain used is *E. coli* C600 (carrying *bla*_NDM‐1_). Data are expressed as log10 percent conjugation frequency (mean ± SD). (***p < 0.001).

Our analysis revealed a significant inverse relationship between virulence and antimicrobial resistance in CC23‐K1 strains. Single‐factor analysis indicated that strains encoding *bla*_NDM_ or *bla*_KPC_ genes exhibited significant genetic reductions in regions coding capsule synthesis (*cps*), O‐antigen synthesis (*ops*), salmochelin (*iro*), and mucoid phenotype regulator (*rmp*) (Fisher exact test *p* < 0.01; Figure S8A–H). Combining gene disruption and deletion, we found that over 60% of the isolates carrying *bla*_KPC‐2_ (*p* < 0.001), *bla*_NDM‐1_ (*p* < 0.001), *bla*_NDM‐5_ (*p* < 0.001), or multiple carbapenemase genes (*p* < 0.05) showed partial or complete deletion of key virulence genes. In contrast, isolates with *bla*_OXA‐48_‐like, *bla*_IMP‐26_, or *bla*_VIM‐1_ genes maintained virulence gene integrity comparable to CSKPs (Figure 4A). To address potential sampling bias, we stratified all CC23‐K1 isolates by geographic origin, project, and isolation source, consistently observing the inverse relationship between virulence genes and *bla*_NDM_‐/*bla*_KPC_ genes (*p* < 0.05) across various subpopulations (Table S6).

We further employed ancestral state reconstruction analysis of all virulence factors to minimize sampling bias. This phylogenetic approach confirmed the inverse relationship between the presence of *cps*, *ops*, *iro*, and *rmp* genes and the acquisition of *bla*_KPC_ or *bla*_NDM_ genes (all *p* < 0.01; Tables S7 and S8). Importantly, our evolutionary analysis suggests that virulence gene reduction possibly preceded carbapenemase gene acquisition, as indicated by the differential increase in evolutionary events. For example, the frequency of resistance gene acquisition increased from 2.31% to 6.33% (2.7× increase) in the ancestral nodes lacking O antigens (Table S7), which substantially exceeded the increased frequency of O antigen deletions (from 7.98% to 14.02%, 1.8× increase) in ST23 CRKPs (Table S8). These patterns could reflect either direct trade‐offs or parallel adaptation to different selective pressures in clinical environments.

We further examined CRKP clusters, groups of ≥3 genetically related isolates, compared to singletons. When analyzing only strains outside these clusters to control for potential epidemiological bias, we still observed reduced virulence gene integrity in isolates carrying *bla*_KPC‐2_ (*p* < 0.001), *bla*_NDM‐1_ (*p* < 0.001), or multiple carbapenemase genes (*p* < 0.05), confirming our initial observations (Figure 4B).

CRKP clusters, representing strains recovered from multiple patients, likely represent more successful lineages than the singletons that leave no observable descendants. Based on the hypothesis that virulence‐resistance trade‐offs result from natural selection, we predicted more pronounced trade‐offs in the CRKP clusters. Our analysis confirmed this prediction, revealing more extensive virulence gene deletions in CRKP clusters, particularly those carrying *bla*_KPC‐2_ and *bla*_NDM‐5_ (all *p* < 0.05; Figure S8I). Seven CRKP clusters completely lacked isolates with intact virulence gene sets, while four additional clusters exhibited significant virulence gene deletions (Figure 4C). The *bla*_OXA_‐associated CRKPs maintained most virulence genes, potentially due to their reduced activity against carbapenems and cephalosporins compared to *bla*_KPC_ and *bla*_NDM_ (Figure 4D), resulting in lower evolutionary costs for simultaneously maintaining both resistance and virulence mechanisms.

### Universal presence of virulence‐resistance trade‐off in *K. pneumoniae*

Analysis of 80,300 publicly available *K. pneumoniae* genomes identified 14,600 non‐CC23 hypervirulent *K. pneumoniae* (hvKPs) with virulence scores of ≥3 by Kleborate, predominantly from ST11 (4699), ST147 (1203), and ST15 (580). Over three‐quarters (11,117; 76%) of them also encoded carbapenemase (hvCRKPs). Most hvKPs encoded *iuc* (98%), *ybt* (77%), and *rmpA2* (71%) (Figure 4E and Table S9). However, *rmp*, *iro*, and *clb* appeared in 45%, 58%, and 15% of hvCSKPs, respectively, compared to only 24%, 5%, and 2% of hvCRKPs (all *p* < 1 × 10^−6^), consistent with patterns observed CC23‐K1. ST11‐L1 (the Chinese clade of ST11, defined by capsular types K47 and K64), responsible for most hospital‐acquired infections in China, also showed a low prevalence of these genes (35%, 5%, and 0.3%, respectively) (Figure S8J,K) [15].

### Experimental validation of the virulence‐resistance trade‐off

In vivo experiments using a murine model demonstrated the association between the virulence and the completeness of the key virulence factors, specifically K‐antigen, *iro*, and *rmp*, which we predicted above to be frequently deleted in resistance strains. Strains with any deletion in these genes exhibited significantly reduced virulence, failing to induce high mortality in the mice within 14 days even at high inoculum levels (5 × 10⁵ CFU) (Figure 4F, Figure S9). In contrast, fully virulent CC23‐K1 strains caused 100% mortality under identical conditions.

Examination of gene deletions' impact on capsule production revealed that *wcaJ* deletion, the initial glycosyltransferase gene in capsular polysaccharide synthesis, completely abolished capsule production (Figure 4G). Deletion of *rmpA* reduced capsule production by half (20 μg/OD vs. 40 μg/OD) and significantly decreased in vivo virulence. Conversely, the deletion of *wzi*, which involved attaching capsular polysaccharide to the outer membrane, dramatically reduced the bacterial surface attached capsule (6 μg/OD) but minimally impacted virulence, challenging assumptions about the direct correlation between capsule production and virulence.

Additionally, we observed that high levels of capsule production inhibited plasmid transfer. Transfer frequencies of a *bla*_NDM‐1_‐carrying plasmid into 6113, an ST23 strain, increased by 10‐fold after *wcaJ* knockout (Figure 4H). Additionally, enzymatic digestion of capsular polysaccharides increased plasmid transfer by fourfold, demonstrating the physical barrier effects of the capsule. These findings support an evolutionary trade‐off between resistance and virulence, where strains prioritizing virulence mechanisms may sacrifice resistance gene acquisition capability.

### Genomic diversity and selection patterns

**Figure 5:**
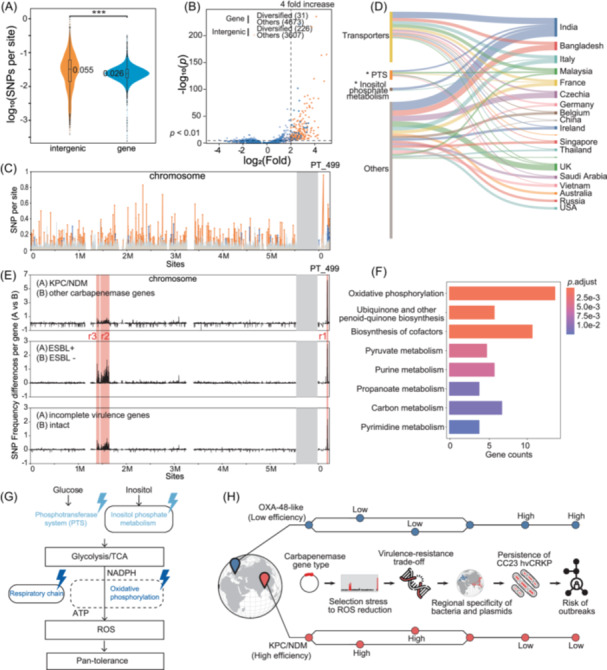
Genomic diversity, selection patterns, and the virulence‐resistance trade‐off model in *K. pneumoniae* CC23‐K1. (A) Comparison of mutation rates between intergenic regions (orange) and coding genes (blue) in the CC23‐K1 core genome. Intergenic regions show significantly higher mutation rates (0.055 single‐nucleotide polymorphisms (SNPs) per site) compared to coding regions (0.026) (*p* < 0.001). (B) Volcano plot showing highly mutated regions (HMRs) identified by Fisher's exact test with Bonferroni correction (*p* < 0.01). Orange points represent intergenic regions (226 diversified, 307 others) and blue points represent genes (31 diversified, 4573 others). The dashed line in *x* = 4 indicates a fourfold increase in mutation rate. (C) Distribution of SNPs across the CC23‐K1 chromosome and the virulence plasmid PT_499. Orange bars represent intergenic mutations, and blue bars represent mutations in coding regions. (D) Sankey diagram showing the geographic distribution of mutations in HMRs with significant country‐specific patterns. Functional categories of genes were on the left with countries shown on the right. The two enriched pathways were marked with “*”, and with preferential mutations in inositol metabolism genes in India and phosphotransferase systems (PTSs) in Asian countries, by analysis of variance (ANOVA) tests with Bonferroni correction (*p* < 0.01). (E) Comparative analysis of SNP frequency differences per gene in three independent comparisons: isolates carrying (A) *bla*_KPC_/*bla*_NDM_ versus (B) other carbapenemases (top), (A) ESBL‐positive versus (B) ESBL‐negative isolates (middle), and isolates with (A) incomplete versus (B) intact virulence genes (bottom). Three genomic regions (r1, r2, r3) consistently show significant differential SNP patterns across all three comparisons (Tukey's honestly significant difference (HSD) post hoc test, *p* < 0.01). (F) Functional enrichment of genes preferentially mutated in isolates carrying *bla*_KPC_/*bla*_NDM_. (G) Proposed metabolic pathways associated with the four enriched pathways. Mutations in PTS and inositol metabolism may reduce the flow of central metabolism, while mutations in menaquinone synthesis and oxidative phosphorylation may reduce the flow of electron transport chain (ETC), both resulting in the reduction of reactive oxygen species (ROS) and pan‐tolerance. (H) Proposed balance model explaining the virulence‐resistance trade‐off in *K. pneumoniae* CC23‐K1 across geographic regions. The model illustrates how different carbapenemase genes (*bla*_OXA‐48_‐like with low efficiency vs. *bla*_KPC_/*bla*_NDM_ with high efficiency) influence the virulence‐resistance balance, leading to regional differences in the prevalence of hvCRKP and associated outbreak risks.

We observed 144,153 mutations in the 4.9 Mb core genome of CC23‐K1 after excluding recombination events, resulting in an average mutation frequency of 0.029 per site. Notably, intergenic regions, genomic intervals between annotated protein‐coding genes that account for only ~10% of the core genome, experienced significantly more mutations compared to the coding regions, contributing to 20% of the mutations (Figure 5A).

Using Fisher's exact test with Bonferroni correction, we identified 226 intergenic regions and 31 genes as highly mutated regions (HMRs) (*p* < 0.01) (Figure 5B,C and Table S10). This enrichment of intergenic HMRs appears distinctive to CC23‐K1 because comparable analysis of the *K. pneumoniae* ST11 lineage showed similar numbers of highly mutated genes and intergenic regions (73 vs. 88, Figure S10A and Table S11).

Functional analysis of genes in or near HMRs revealed enrichment in inositol metabolism and phosphotransferase systems (PTS), pathways critical for carbon source uptakes, upstream of the glycolysis and tricarboxylic acid cycle (TCA) (Figure S10B,C). This includes the *iolCDE* gene cluster, essential for inositol utilization and osmotic and oxidative stress responses, and genes encoding phosphotransferases for Glucose (*ptsG*), β‐Glucoside (*ascF*), Trehalose (*treB*), Cellobiose (*celAB*), and the others, which are associated with cAMP‐dependent persistence and virulence regulation [16].

One‐way ANOVA demonstrated significant geographic variation in mutation frequencies for approximately half (113/257) of identified HMRs (Figure 5D), with distinct mutation enrichments in specific countries. Notably, three of four regions involving inositol metabolism showed preferential mutation in India, and half (6/12) of the phosphotransferases were preferentially mutated in Asian countries, possibly reflecting metabolic pathway adaptation to local conditions.

### The influence of virulence‐resistance trade‐off in the core genome

ANOVA analyses identified three core genomic regions with significantly higher mutation rates in CRKPs carrying *bla*_KPC_ and *bla*_NDM_ variants compared to those with other carbapenemase genes (Figure 5E). The same regions, designated as the resistance‐associated regions (RARs), also differentiated isolates carrying extended‐spectrum beta‐lactamase (ESBL) genes from those without and isolates with intact versus compromised virulence gene sets, underscoring their importance in the evolution of resistance.

Functional analysis of these three regions revealed enrichments of genes associated with oxidative phosphorylation and menaquinone biosynthesis (Figure 5H), particularly the *nuoABCEF* genes responsible for NADH:quinone oxidoreductase (complex I) activity, and *menBCDEFH* genes responsible for menaquinone biosynthesis (Figure S10D). Both pathways are involved in the electron transport chain (ETC), driving the synthesis of adenosine triphosphate (ATP) and producing reactive oxygen species (ROS) as a byproduct.

Collectively, the four enriched pathways (inositol metabolism, PTS, oxidative phosphorylation, and menaquinone biosynthesis) were all associated with the central metabolic processes. Genetic variations might have altered their functions and reduced ETC flow, resulting in lower ROS levels and better tolerance to various stresses [17] (Figure 5G).

### Proposed virulence‐resistance balance model

The virulence‐resistance trade‐off exhibits distinct regional patterns, suggesting that local ecological and epidemiological factors shape the evolutionary dynamics of carbapenem‐resistant *K. pneumoniae* (CRKP) (Figure 5H). In several European regions, the predominance of *bla*_OXA‐48_‐type carbapenemases may be associated with a genomic context that permits retention of virulence determinants, potentially facilitating the emergence of hypervirulent CRKP (hvCRKP). In contrast, CRKP strains circulating in many Asian regions more frequently harbor high‐efficiency carbapenemases such as *bla*_KPC_ or *bla*_NDM_, which are often accompanied by reduced virulence gene content.

## DISCUSSION

The global emergence of hvCRKP represents a critical threat to public health. Our comprehensive analysis of the CC23‐K1 lineage, spanning genomic, geographic, and experimental approaches, reveals fundamental insights into the genomic plasticity, regional segregation, and hypervirulence‐resistance trade‐offs that have substantially influenced the evolutionary dynamics of this high‐risk clone.

The phylogeographic reconstruction of CC23‐K1 demonstrates a lineage that, while globally distributed, exhibits striking geographic compartmentalization with distance‐driven transmission patterns (Figure 1C). This pattern contrasts with the pandemic spread of other multidrug‐resistant clones such as *K. pneumoniae* ST258/ST11, which achieved dominance through rapid clonal expansion [15, 18]. The accumulation of over 2000 international CC23 genomes, contributed by all researchers worldwide (e.g., those from Ireland and Singapore [5, 19]) allowed us to reveal the limited inter‐continental transmission, evidenced by the strong negative correlation between transmission frequency and geographic distance. This suggests that geographic barriers and localized selection pressures govern dissemination, similar to *Streptococcus pneumoniae* in South Africa [20].

Molecular dating places CC23‐K1's origin in the mid‐19th century, with rapid population expansion in the 1940s coinciding with post‐World War II globalization [9, 21]. This historical context aligns with similar expansion patterns in other successful global pathogens [14, 22].

A key finding is the transient nature of carbapenem resistance in CC23‐K1. Despite acquiring carbapenemase genes at least 130 times (Figure 1B), resistance rarely stabilized, with 95% of carbapenem‐resistant clusters co‐occurring with susceptible strains within narrow genetic distances (Figure S4B). This instability contrasts sharply with lineages like ST11, where carbapenem resistance remains stable for decades [15]. Such instability likely reflects opposing selective forces: carbapenemase plasmids impose fitness costs poorly tolerated by hypervirulent strains in nonselective environments, while antibiotic‐rich settings favor plasmid retention, similar to observations in *Escherichia coli* ST131 [23, 24].

Geographic specificity further shapes resistance patterns, with regional prevalence of specific carbapenemases reflecting local plasmid ecology (Figure 3A,B) and selection pressures [25]. This geographical association of carbapenemases and their host plasmids is consistent with patterns previously documented across the broader *K. pneumoniae* population and other pathogens [2, 26, 27]. Horizontal transfer appears to dominate plasmid spread, as evidenced by the minimal contribution of bacterial genetic context to plasmid distribution.

Perhaps the most significant finding is the potential trade‐off between hypervirulence and carbapenem resistance. Strains carrying *bla*_KPC‐2_ or *bla*_NDM_ frequently lost virulence genes, while *bla*_OXA‐48_‐ or *bla*_VIM_‐positive strains retained hypervirulence (Figure 4A). This pattern extends beyond CC23 to other hypervirulent lineages, suggesting a fundamental evolutionary constraint, a phenomenon also documented in other pathogens [28]. In contrast, *bla*_OXA‐48_'s lower enzymatic activity on carbapenems may impose a reduced metabolic burden (Figure 4D), allowing compatibility with virulence traits [29].

Our analyses and experiments both revealed that capsule production physically impedes plasmid conjugation (Figure 4H), providing a mechanistic basis for this trade‐off. While differing from the scoring system in Kleborate, the most important virulence genes we identified align closely with earlier evaluations and highlight the importance of gene integrity and extracellular polysaccharides in bacterial infections [15, 30, 31]. This finding also aligns with previous studies in *Klebsiella*, which suggested the role of phage selection, and other encapsulated pathogens where capsule expression negatively correlates with horizontal gene transfer [32, 33, 34, 35].

CC23‐K1 exhibits exceptional genomic diversity, with doubled mutation frequency in intergenic regions compared to genes (Figure 5A‐C). This pattern, distinct from ST11, suggests that CC23‐K1 prioritizes regulatory evolution over protein‐coding changes to maintain metabolic flexibility, a strategy observed in other niche‐adaptive pathogens [36]. Some of such enriched mutations, associated with the virulence‐resistance balance, likely have become fixed in the Asian populations, fundamentally reshaping CC23‐K1's evolution.

Functional analysis linked these mutations to pathways critical for environmental adaptation, including inositol metabolism (e.g., *iolCDE*), PTS (*ptsG, ascF, treB, celAB*, etc.), oxidative phosphorylation (*nuoABCEF*), and menaquinone biosynthesis (*menBCDEFH*) (Figure 5G). These pathways form a cascade from carbon source uptake through glycolysis and the TCA cycle to ETC function. While highly efficient and essential, this process generates substantial ROS, which mediates cell death under environmental stressors such as antimicrobial exposure or elevated temperatures. Partial deletion of either PTS or ETC systems can reduce ROS production and substantially increase bacterial pan‐tolerance to many stresses [37, 38].

Notably, the pathways enriched in HMRs, characterized by massive transient mutations, are located upstream of central metabolism, which generates not only energy but also numerous intermediate metabolites essential for other biological pathways. In contrast, downstream mutations (RARs) become fixed with minimal disruption to essential processes. This strategy is possibly associated with the successful fixation of alterations in these regions.

Furthermore, these mutated pathways participate in additional biological processes beyond energy metabolism. For example, inositol metabolism has been linked to stress response and osmotic adaptation in gram‐negative bacteria, potentially enhancing survival in healthcare environments with frequent disinfectant exposure [39]. Similarly, PTS components have been linked to biofilm formation and control of virulence gene expression in *K. pneumoniae*, suggesting multifaceted advantages conferred by these mutations [40, 41].

While our comparative genomic data suggest that mutations in ETC‐associated pathways may be associated with reduced ROS production and enhanced stress tolerance, this remains a hypothesis in the absence of direct functional validation (e.g., ROS quantification assays), and further experimental work is required before definitive conclusions can be drawn.

The virulence‐resistance trade‐off manifests differently across geographic regions, possibly influenced by antimicrobial consumption patterns (Figure 5H). European countries with modest carbapenem usage (2.6 defined daily dose per 1000 inhabitants per day (DID), 13% of total antimicrobial use) show predominance of *bla*_OXA‐48_‐type carbapenemases compatible with virulence maintenance [42]. Asian countries with higher consumption (4‐6 DID, 20%–30% of total antimicrobial use) favor high‐efficiency enzymes incompatible with complete hypervirulence determinants [43, 44]. This geographic divergence in antimicrobial stewardship may have created distinct evolutionary trajectories for hvCRKP.

Our study has limitations. Retrospective sampling underrepresents regions like Africa and South America, potentially biasing transmission inferences and masking rare but epidemiologically significant events, such as transcontinental transmissions or alternative plasmid lineages. For example, a recent study from Chile described the emergence of a carbapenem‐resistant hypervirulent ST23 *K. pneumoniae* strain carrying a dual‐carbapenemase plasmid with high transmissibility in a hypervirulent background, highlighting the urgent need to expand surveillance efforts in the Global South. Long‐term surveillance in underrepresented regions is critical to refine models of CC23‐K1's global spread [45].

Importantly, while our in vivo murine experiments and genomic correlations suggest a linkage between carbapenemase acquisition and virulence‐gene loss, direct mechanistic validation remains lacking and requires further investigation. The clinical isolates used are not isogenic for resistance or virulence loci, background genomic differences could confound the observed trade‐off. Future work employing isogenic strain pairs and controlled plasmid‐transfer assays will be critical to establish causality and dissect plasmid‐versus chromosomal contributions to virulence attenuation.

## CONCLUSION

In summary, CC23‐K1's evolutionary success hinges on balancing genomic plasticity, geographic adaptation, and an intriguing trade‐off between virulence and resistance. Unlike pandemic clones that optimize transmission through genetic stability, CC23‐K1 thrives through mutational agility and transient gene acquisition. This strategy permits niche‐specific adaptation but limits global dominance, a paradox offering both challenges and opportunities for control. Future surveillance pipelines could be enhanced by integrating machine learning and deep learning approaches, which have shown promise in identifying high‐risk clones, predicting antimicrobial resistance, and uncovering cryptic epidemiological trends using partial genomic or phenotypic data. By tailoring surveillance to regional resistance patterns and leveraging the resistance‐virulence trade‐off in treatment strategies, we can mitigate the potential threat of this elusive pathogen.

## METHODS

### Sample collection and genome sequencing

We collected 151 *K. pneumoniae* CC23 isolates from six provinces in China between 1999 and 2024. These isolates were obtained from clinical samples in multiple healthcare facilities, including a teaching hospital in Hangzhou, Zhejiang, where we conducted longitudinal surveillance over 30 years. Clinical isolates were identified as *K. pneumoniae* using standard microbiological methods, including MALDI‐TOF mass spectrometry and biochemical testing.

Genomic DNA was extracted using the QIAamp DNA Mini Kit (Qiagen) according to the manufacturer's instructions. Library preparation was performed using the Nextera XT DNA Library Preparation Kit (Illumina), and whole‐genome sequencing was conducted on the Illumina NovaSeq 6000 platform with 250‐bp paired‐end reads.

### Genome assembly and annotation

Raw sequencing reads were quality‐filtered using Trimmomatic v0.40 to remove adapter sequences and low‐quality bases (quality score < 20) [46]. *de novo* assembly was performed using SPAdes v3.15.0 with default parameters. The assembled genomes were annotated using Prokka v1.14.5 [47, 48]. All newly sequenced genomes were deposited in https://github.com/puff0916/ST23_kle.

To create a comprehensive data set, we combined our 151 sequenced genomes with 2412 publicly available *K. pneumoniae* CC23 genomes from the NCBI GenBank, resulting in a total of 2563 genomes from 62 countries spanning from 1932 to 2024. Publicly available genomes were downloaded and processed using the same assembly and annotation pipeline as our sequenced isolates to ensure consistency. The quality of our assemblies and those from public databases were all evaluated using Kleborate v3.1.3, keeping only assemblies with N50s of ≥10 Kb and total sizes between 5 and 6.5 MB [30]. The sublineages and clonal groups of the CC23 genomes were predicted using the cgLINcode scheme hosted in https://pathogen.watch/ [13].

### Identification of carbapenem resistance genes and plasmids

Resistance genes, virulence genes, and Institut Pasteur 7‐locus MLST STs were all predicted using Kleborate v3.1.3 [30]. Plasmids were predicted and genotyped using KleTy, which identifies plasmid replicons and characterizes plasmid types based on gene content and sequence similarity [2]. Plasmid phylogenies were constructed using core genome alignments and maximum‐likelihood methods as described above.

Geographic association of plasmids was quantified using Theil's uncertainty coefficient (U). For each plasmid type, we constructed a contingency table of plasmid presence/absence across countries and bacterial clades. Theil's U was calculated to measure the proportion of uncertainty in plasmid distribution explained by either geographic origin or bacterial clade.

### Phylogenetic and population structure analysis

To reconstruct the evolutionary history of CC23, we performed a core genome phylogenetic analysis using the EToKi package [49]. EToKi aligned all CC23 genomes onto one single reference (GCA_027594925) using minimap2‐2.28 and had recombination regions identified and removed using RecHMM [50, 51]. A maximum‐likelihood phylogenetic tree was constructed using IQ‐TREE2 v.2.4.0 [52] under the GTR + GAMMA model. The acquisition/deletion of carbapenemase genes and virulence genes were all inferred using stochastic character mapping implemented in TreeTime v0.11.0 [53].

For the analysis of the CC23‐K1 lineage, we extracted the 2220 genomes belonging to this lineage based on the initial phylogenetic analysis and generated a separate recombination‐filtered alignment. The CC23‐K1 phylogeny was constructed using the same approach as described above. The major clades in CC23‐K1 were identified using rhierBAPs (https://github.com/gtonkinhill/rhierbaps). We ran the analysis with the following parameters: a maximum number of clusters set to 20, allowing two levels of clustering, and using 10 independent random starting configurations to improve the robustness of cluster assignments. We inspected the results visually and kept only the first level, as the second level was inconsistent with the phylogeny.

Pairwise SNP distances were calculated for all isolate pairs, and a distance threshold of 93 SNPs was determined as the optimal threshold that separates CRKPs within the same cluster from those between clusters using Fisher's exact test. This threshold was used to define genetic modules, with isolates connected if they differed by fewer than 93 SNPs. Network visualization was performed using Gephi v0.11.0 (https://gephi.org/).

### Temporal analysis and phylogeography

To investigate the temporal dynamics of CC23‐K1, the temporal signal was evaluated in the data set using TempEst v1.5.3 [54]. We performed time‐calibrated phylogenetic analysis using TreeTime v0.11.0 [53] with a relaxed molecular clock model, which automatically identified and excluded outliers with excessively long branches. The effective population size was estimated using the skyline method with 20 time intervals. Additionally, we performed Bayesian evolutionary analyses using BEAST v2.6.8 [55]. Due to the large data set size, a subsampling strategy was implemented as previously described [51], dividing the CC23‐K1 strains into four equal subgroups for parallel inference.

For each subgroup, we evaluated four alternative models: (1) strict clock with constant population size, (2) strict clock with extended Bayesian skyline plot, (3) relaxed clock with constant population size, and (4) relaxed clock with extended Bayesian skyline plot. Model selection was performed using nested sampling [56]. The optimal model, relaxed clock with extended Bayesian skyline plot, was subjected to a long run of 10 billion generations. The first 10% of samples were discarded as burn‐in. Convergence was assessed using Tracer v1.7.1, ensuring effective sample sizes >200 for all parameters.

Geographic transmission patterns were inferred using stochastic character mapping implemented in TreeTime. To account for sampling bias, we performed 100 random down‐samplings as previously described, selecting up to 10 genomes per country for each iteration [14]. Transmission events were extracted from the ancestral state reconstructions, and the transmission network was visualized using Gephi. Countries were clustered into modules using the Louvain community detection algorithm.

### Species‐scale analysis of *K. pneumoniae*

To contextualize CC23‐K1 within the broader *K. pneumoniae* population, we analyzed 80,300 publicly available *K. pneumoniae* genomes from NCBI GenBank. Species identification was confirmed using Kleborate v3.1.3, which also assigned sequence types and detected antimicrobial resistance and virulence genes.

Hypervirulent *K. pneumoniae* (hvKP) isolates were identified based on their virulence scores of ≥3, which was associated with the presence of *iuc* loci. Some *iuc* loci are treated as deleted, due to the disruption of their coding frames by mutations or insertions/deletions. Carbapenemase‐producing isolates were identified based on the presence of associated genes.

For global distribution analysis, isolates were categorized by country, continent, and sequence type. Statistical comparisons of virulence gene frequencies between carbapenem‐resistant and carbapenem‐susceptible isolates were performed using Fisher's exact test, with Bonferroni correction for multiple comparisons.

### Murine infection model

Eight‐week‐old female C57BL/6 J mice were used for the virulence assessment in the present study. Mice were inoculated intraperitoneally with 5 × 10^5^ colony‐forming units (CFUs) of *K. pneumoniae* bacterial suspension in a volume of 200 µL. After infection, the animals were monitored daily for 14 days for clinical signs of illness, including weight loss, reduced mobility, and abnormal respiration. Humane endpoints were strictly implemented to minimize animal suffering, such as weight loss exceeding 20% of baseline, persistent immobility, or labored breathing, were humanely euthanized before the endpoint of the study. Survival analysis was performed on 6 mice per strain. The survival rate was analyzed by the Kaplan–Meier method with a log‐rank test; the difference was considered statistically significant at *p* < 0.05.

### Construction of *K. pneumoniae* 6113 ΔCPS mutant

The *K. pneumoniae* 6113 Δ*wcaj* mutant (ΔCPS) was constructed using the λ Red‐dependent recombination system and the FLP/FRT system. First, a donor DNA fragment consisting of 500 bp upstream and downstream of the *wcaJ* gene, along with a hygromycin‐resistant gene, was constructed via Gibson assembly. The hygromycin‐resistant gene flanked by FRT sites was PCR‐amplified from the pUC19‐Hph plasmid. The pKOBEG plasmid, carrying the λ Red operon and an apramycin‐resistant gene, was introduced into the 6113 strain by electroporation. After induction with arabinose, the donor DNA was electroporated into the 6113 strain, replacing the *wcaJ* gene with the hygromycin resistance gene. Mutants were selected on LB agar plates containing apramycin and hygromycin and confirmed by PCR and sequencing. The pKOBEG plasmid was removed by growing the mutants at 42°C.

### Conjugation assays

Conjugation assays were performed as previously reported [57]. The donor strain, *E. coli* C600 (harboring *bla*_NDM‐1_), and the recipient strain (KL1 type *K. pneumoniae* 6113 WT strain and ΔCPS mutant) were cultured overnight and then subsequently subcultured at a 1:100 dilution in LB medium at 37°C to reach the OD_600_ of 0.7. For the depolymerase‐treated group, *K. pneumoniae* 6113 strain was treated with K1 capsular polysaccharide depolymerase (20 μg/mL) for 30 min at 37°C before mixing with the donor strain. Equal volumes of donor and recipient cells were mixed at a 1:1 ratio, washed with PBS buffer, and resuspended in 50 μL of 10 mM MgSO₄. The cell suspension was then seeded on a 0.45 μm pore‐size nitrocellulose filter on the surface of an LB agar plate and incubated at 37°C for 7 h. After incubation, the cells were recovered from the filters, resuspended in PBS buffer, and serially diluted. The bacterial suspensions were plated on MIAC agar with or without 4 μg/mL meropenem to determine the conjugation frequency.

### Competitive ELISA for capsule production

Competitive ELISA was performed as previously reported [58]. Rabbit antisera against the K1 polysaccharide were absorbed by incubation with *K. pneumoniae* 6113 Δ*wcaJ* mutant to remove OPS and core‐specific antibodies, which may cross‐react in the iELISA. *K. pneumoniae* strains with OD 1.5 were fixed with 4% paraformaldehyde and then serially diluted 2.5‐fold and incubated with the adsorbed K1 antisera at 37°C for 2 h. The iELISA plate was coated with purified K1 polysaccharide (5.0 μg/mL) and methylated human serum albumin (1.0 μg/mL) at 37°C for 3 h. The washed plate was then blocked by the 5% calf serum (150 μL/well) at 37°C for 1 h and washed twice. Then, the supernatant of K1 antisera incubated with different diluted *K. pneumoniae* strains was added to the iELISA plate at 4°C overnight. The washed plate was then incubated with AP goat anti‐rabbit immunoglobulin G (1/20,000 dilution, 100 μL/well) at 25°C for 2 h, washed 5 times, and incubated with chromogenic reagent (100 μL/well) at 25°C for 2 h. After being stopped by 3 M NaOH, A_405_ was recorded. K1 capsular polysaccharide (1860 μg/mL), 2.5‐fold serial dilution, and incubation with adsorbed K1 antisera for iELISA to construct a standard curve. The amount of K1 capsules of *K. pneumoniae* strains was calculated from the standard curve.

### Identification of HMRs

To identify genomic regions under diversifying selection, we calculated the frequency of mutations across all protein‐coding genes and intergenic regions in the CC23‐K1 lineage. Mutations introduced by recombination events were excluded from this analysis. For each gene and intergenic region, we performed a Fisher's exact test to compare the observed mutation frequency with the frequencies in other regions. Statistical significance was assessed with a Bonferroni correction for multiple testing.

HMRs were defined as genes or intergenic regions with significantly higher mutation frequencies (adjusted *p*‐value < 0.01). Geographic variation in mutation frequencies was assessed using one‐way ANOVA, with countries grouped by continent to ensure sufficient sample sizes.

For comparative analysis with the ST11 lineage, we applied the same approach to a data set of 1200 ST11 genomes collected from our previous studies.

### Identification of RARs

For the identification of RARs, we performed three independent comparative analyses using one‐way with Tukey's HSD post hoc test:

1. Isolates carrying *bla*_KPC_/*bla*_NDM_ carbapenemases (Group A) versus isolates carrying other carbapenemase types (Group B).
2. ESBL‐positive isolates (Group A) versus ESBL‐negative isolates (Group B).
3. Isolates with incomplete virulence gene sets (Group A) versus isolates with intact virulence gene sets (Group B).

For each comparison, we calculated the mean SNP frequency difference per gene between Group A and Group B. Negative values indicate that Group B has a higher mutation rate than Group A for that particular gene, while positive values indicate Group A has a higher mutation rates. We performed analogous comparisons for ESBL‐positive versus ESBL‐negative strains and for intact versus compromised virulence gene sets. Regions exhibiting a mean difference of ≥0.5 SNPs per gene and *p* < 0.01 (Bonferroni corrected) in all three comparisons were designated as RARs.

### Functional and geographic enrichment analysis

To characterize the functional significance of HMRs, we performed KEGG pathway enrichment analyses using the R package “clusterProfiler” (v4.0.5). Genes located within or adjacent to HMRs were used as input. We considered pathways with adjusted *p*‐values < 0.05 as significantly enriched.

To investigate geographic variation in mutation patterns, we calculated the accumulated variations from the root in each strain and grouped strains by country of isolation. We performed one‐way ANOVA to compare the number of accumulated variations across regions for each HMR. Geographic specificity was determined by comparing mutation frequencies in strains from individual countries to the overall distribution (*p* < 0.01 after Bonferroni correction).

### Statistical analysis

Statistical analyses were performed using R v4.1.0. Differences between groups were assessed using appropriate statistical tests, including Student's *t*‐test and Fisher's exact test. *p*‐values less than 0.05 were considered statistically significant. One‐way ANOVA with Tukey's HSD post hoc test was used for group comparisons. Bonferroni correction was applied for other multiple independent tests.

The correlation between geographic distance and transmission frequency was assessed using linear regression analysis. Geographic distances between countries were calculated as the great‐circle distance between country centroids and log‐transformed before analysis.

## AUTHOR CONTRIBUTIONS

**Yuchen Wu**: Data curation; validation. **Fan Pu**: Formal analysis; visualization; writing—original draft. **Zelin Yan**: Data curation. **Yanyan Zhang**: Writing—original draft; formal analysis. **Kaichao Chen**: Formal analysis; writing—original draft. **Shengkai Li**: Formal analysis; writing—original draft; methodology; software. **Yuezhuo Wang**: Writing—original draft; visualization. **Heyuan Lun**: Investigation. **Tingting Qu**: Investigation. **Jing Wang**: Validation. **Heng Li**: Validation. **Danxia Gu**: Validation. **Sheng Chen**: Validation. **Ping He**: Conceptualization; writing—review and editing; project administration. **Rong Zhang**: Conceptualization; writing—review and editing; supervision; project administration; funding acquisition. **Zhemin Zhou**: Conceptualization; writing—review and editing; supervision; project administration; funding acquisition.

## CONFLICT OF INTEREST STATEMENT

The authors declare no conflicts of interest.

## ETHICS STATEMENT

The study was approved by the Institutional Review Board (IRB) of The Second Affiliated Hospital of Zhejiang University School of Medicine (No. 2024‐1335). Informed consent was waived as this study utilized bacterial isolates and genomic data that were anonymized and did not include any personal or clinical information that could identify individuals. All procedures were conducted in accordance with relevant ethical guidelines and regulations. For experiments involving animal models, strict ethical standards were followed to ensure humane treatment and minimize suffering. The study protocol was reviewed and approved by the Institutional Animal Care and Use Committee (IACUC) of the Soochow University (No. SUDA20241108A01). Animals were monitored closely for signs of distress, and endpoints were predefined to avoid unnecessary suffering. Euthanasia was performed in accordance with established guidelines to ensure a humane and painless process. All animal experiments complied with the ARRIVE guidelines and relevant regulations on the ethical use of animals in research.

## Supporting information


**Figure S1:** Temporal trends and characteristics of CC23‐K1 *K. pneumoniae* isolates.
**Figure S2:** Phylogenetic analysis of CC23 *K. pneumoniae* isolates.
**Figure S3:** Phylogenetic analysis of CC23‐K1 lineage and global distribution of carbapenemases.
**Figure S4:** Genetic relationships between CC23‐K1 isolates.
**Figure S5:** Temporal signal analysis and evolutionary dating of the CC23‐K1 lineage.
**Figure S6:** Geographic transmission patterns of CC23‐K1 *K. pneumoniae*.
**Figure S7:** Geographic clustering of carbapenemase‐carrying plasmids.
**Figure S8:** Distribution of virulence gene integrity across carbapenemase‐producing *K. pneumoniae* isolates.
**Figure S9:** Assessment of *K. pneumoniae* strains virulence by intraperitoneal infection of mice.
**Figure S10:** Analysis of highly mutated regions and metabolic pathway enrichment in *K. pneumoniae* lineages.

**Table S1:** Allelic profiles of STs in CC23 (ST23 and its single locus variants).
**Table S2:** Metadata and genotype of all CC23 (ST23 and single locus variants) genomes.
**Table S3:** Frequencies of carbapenemase genes in each country.
**Table S4:** Number of transmission events and geographic distances of all possible country pairs.
**Table S5:** Taxonomic and ST distribution of the major plasmids.
**Table S6:** Associations between carbapenemase gene carriages and virulence genes stratified based on the country/isolation/project sources of the isolates.
**Table S7:** The frequency of antimicrobial resistance acquisitions in nodes with or without virulence factors based on ancestral state reconstructions.
**Table S8:** The frequency of gene deletion events in nodes with or without carbapenemase genes based on ancestral state reconstructions.
**Table S9:** Genotypes of all potential non‐ST23 hvCRKPs.
**Table S10:** SNP frequencies of all genes and intergenic regions.
**Table S11:** SNP frequencies of all genes and intergenic regions in ST11.

## Data Availability

The data that support the findings of this study are openly available in GenBank BioProject at https://www.ncbi.nlm.nih.gov/bioproject/, reference number PRJNA1236717. All the sequenced data were deposited in NCBI GenBank under BioProject accession: PRJNA1236717 (https://www.ncbi.nlm.nih.gov/bioproject/PRJNA1236717). The assembled genomes were also accessible at: https://github.com/puff0916/ST23_kle. The data and scripts used are saved in GitHub: https://github.com/Zhou-lab-SUDA/Wu_iMeta_ST23_Figure_sources. A detailed list of the sample accession numbers for all samples is available in Table S2. Supplementary materials (figures, tables, graphical abstract, slides, videos, Chinese translated version, and update materials) may be found in the online DOI or iMeta Science http://www.imeta.science/.
