# Supplementary material for: Geographic containment and virulence‐resistance trade‐offs drive the evolution of hypervirulent Klebsiella pneumoniae
Source: Imeta. 2025 Sep 4;4(5):e70077. doi: 10.1002/imt2.70077 (PMC12528002; doi:10.1002/imt2.70077)
Supplement: Supplementary file 1 — Figure S1: Temporal trends and characteristics of CC23‐K1 K. pneumoniae isolates. Figure S2: Phylogenetic analysis of CC23 K. pneumoniae isolates. Figure S3: Phylogenetic analysis of CC23‐K1 lineage and global distribution of carbapenemases. Figure S4: Genetic relationships between CC23‐K1 isolates. Figure S5: Temporal signal analysis and evolutionary dating of the CC23‐K1 lineage. Figure S6: Geographic transmission patterns of CC23‐K1 K. pneumoniae. Figure S7: Geographic clustering of carbapenemase‐carrying plasmids. Figure S8: Distribution of virulence gene integrity across carbapenemase‐producing K. pneumoniae isolates. Figure S9: Assessment of K. pneumoniae strains virulence by intraperitoneal infection of mice. Figure S10: Analysis of highly mutated regions and metabolic pathway enrichment in K. pneumoniae lineages. [file IMT2-4-e70077-s001.docx]

**Supporting information to**

**Geographic containment and virulence-resistance trade-offs drive the evolution of hypervirulent *Klebsiella pneumoniae***

**Running title**: Geographic containment and virulence-resistance trade-offs in hvKP CC23

Yuchen Wu^1#^, Fan Pu^2,3#^, Zelin Yan^1^, Yanyan Zhang^1^, Kaichao Chen^4,5^, Shengkai Li^2,3^, Yuezhuo Wang^2,3^, Heyuan Lun^6^, Tingting Qu^6^, Jing Wang^2,3^, Heng Li^2,3,7^, Danxia Gu^1^, Sheng Chen^4,5^, Ping He^6*^, Rong Zhang^1*^, Zhemin Zhou^2,3,7*^

^1^ Department of Clinical Laboratory, Second Affiliated Hospital of Zhejiang University, School of Medicine, Hangzhou 310009, China.

^2^ The Second Affiliated Hospital of Soochow University, Cancer Institute, Suzhou Medical College, Soochow University, Suzhou 215006, China

^3^ National Center of Technology Innovation for Biopharmaceuticals, Suzhou Biomedical Industry Innovation Center, Suzhou 215519, China

^4^ State Key Lab of Chemical Biology and Drug Discovery and The Department of Food Science and Nutrition, The Hong Kong Polytechnic University, Kowloon 999077, Hong Kong.

^5^ Shenzhen Key Lab for Food Biological Safety Control, The Hong Kong Polytechnic University Shenzhen Research Institute, Shenzhen 518066, China.

^6^ Department of Immunology and Microbiology, Shanghai Jiao Tong University School of Medicine, Shanghai 200030, China.

^7^ National Key Laboratory of Intelligent Tracking and Forecasting for Infectious Diseases, National Institute for Communicable Disease Control and Prevention, Chinese Center for Disease Control and Prevention, Beijing 102206, China

^#^These authors contributed equally: Yuchen Wu, Fan Pu

*Correspondence: [hpatsh@sjtu.edu.cn](mailto:hpatsh@sjtu.edu.cn) (Ping He), [zhang-rong@zju.edu.cn](mailto:zhang-rong@zju.edu.cn) (Rong Zhang), [zmzhou@suda.edu.cn](mailto:zmzhou@suda.edu.cn) (Zhemin Zhou).

**

****Figure S1** **Temporal trends and characteristics of CC23-K1 *K. pneumoniae* isolates.** (A) Longitudinal surveillance showing the prevalence of CC23-K1 among clinical isolates from a teaching hospital in Hangzhou, Zhejiang, from 1994 to 2024. The purple line represents the percentage of CC23-K1 in CSKP isolates, while the red line shows the percentage in CRKP isolates. A notable peak of CC23-K1 in CRKP occurred between 2004-2008, reaching approximately 15%, followed by a significant decline. In contrast, CC23-K1 maintained a relatively higher presence in CSKP isolates throughout the study period, with current levels around 9.5%. (B) Sankey diagram illustrating the distribution of 150 CC23-K1 isolates collected from China between 1999 and 2024. The diagram links isolation year (left), specimen source (middle), carbapenem resistance status (CSKP/CRKP), and carbapenemase gene types (right).

**
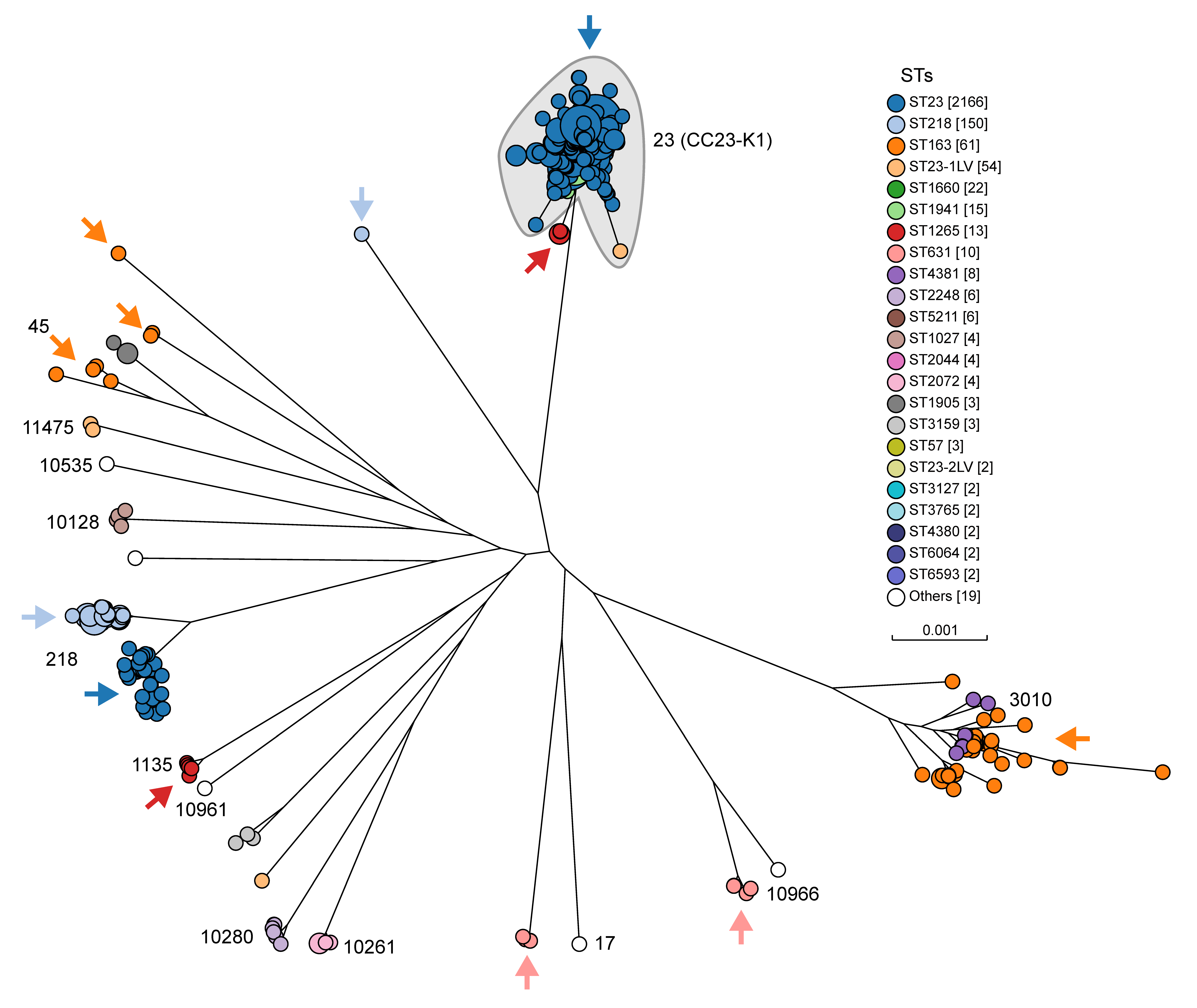
Figure S2 Phylogenetic analysis of CC23 *K. pneumoniae* isolates.** This phylogenetic tree illustrates the polyphyletic nature of the CC23 complex of *K. pneumoniae*, demonstrating that it comprises at least 40 sequence types (STs) distributed across 22 distinct phylogenetic branches. Sublineages, predicted using cgLINcodes in https://pathogen.watch/, were shown nearby the associated branches. The largest cluster, designated CC23-K1 (highlighted in gray at the top), contains the majority of isolates and predominantly consists of ST23 strains (*n* = 2166, shown in dark blue). A secondary notable cluster, labeled CC23-K57 (bottom left), represents another distinct phylogenetic branch primarily comprising ST23 isolates but with a different capsular type. Colored arrows (blue, red, orange, and pink) highlight polyphyletic STs that are incompatible with the phylogeny.



**Figure S3 Phylogenetic analysis of CC23-K1 lineage and global distribution of carbapenemases.** (A) Maximum likelihood phylogenetic tree of the CC23-K1 lineage after removal of recombination regions, showing the seven major clades (A-G) that comprise 93.6% (2077/2220) of CC23-K1 isolates. Each clade is represented by a different color, with the number of isolates per clade indicated in brackets. CRKP clusters are labeled according to their clade designation and predominant carbapenemase gene and highlighted in red. (B) Bubble chart depicting the geographic distribution of carbapenemase genes in CC23-K1 isolates across 22 countries. The size of each bubble corresponds to the relative frequency of each carbapenemase type within a particular country.



**Figure S4 Genetic relationships between CC23-K1 isolates.** (A) Distribution of pairwise SNP distances between CC23-K1 CRKP isolates. The dashed line at 93 SNPs, represents the threshold used to define genetic clusters, with isolates differing by fewer than 93 SNPs (red) considered to be closely related. This threshold was determined based on Fisher’s exact test. (B) Network visualization of CC23-K1 isolates based on the 93 SNP threshold. Each node represents an isolate, and edges connect isolates with fewer than 93 SNPs difference. Colored nodes indicate different carbapenemase types as shown in the legend, while pink nodes represent CSKP. Numbers indicate major cluster identifiers. (C) Composition of large clusters ( ≥ 3 isolates) showing the frequency of different isolate types within each cluster. Colored bars represent different carbapenemase-producing isolates, and pink bars represent CSKP isolates. The black line shows the percentage of CRKPs in each cluster.



**Figure S5 Temporal signal analysis and evolutionary dating of the CC23-K1 lineage.** (A) Root-to-tip distance analysis of CC23-K1 isolates, showing the correlation between genetic divergence and isolation year. The linear regression line (R = 0.17) indicates a significant temporal signal in the data, with isolates collected more recently showing greater genetic divergence from the root. (B) Substitution rate estimates for the CC23-K1 lineage (red) compared with 10 randomly permutated datasets (black). The median rate is indicated by the red point, with error bars representing the 95% confidence interval. The actual data is significantly greater than random simulations. (C) R values from the root-to-tip regression analyses. The higher value (highlighted in red) represents the actual data, while the black points show values from randomized data, validating the significance of the temporal signal. (D) Skyline plot showing the comparable posterior distribution of the time of most recent common ancestor (tMRCA) for the four independent runs of random subsets in (E-H). (E-H) BEAST2 analysis results from four independent runs with random subsets (s1-s4) of CC23-K1 isolates. Each panel shows the time-calibrated phylogenetic tree with branches colored according to the estimated substitution rate. The estimated time to the most recent common ancestor (tMRCA) for each subset is shown at the base of each tree, with 95% confidence intervals in parentheses. The consistent dating results (ranging from 1834 to 1888) across independent analyses corroborate the estimated origin of the CC23-K1 lineage in the mid-19th century.

**

Figure S6 Geographic transmission patterns of CC23-K1 *K. pneumoniae*.** (A) Sankey diagram showing inferred international transmission events based on the original complete dataset. The width of flows represents transmission frequency between countries. (B) Sankey diagram showing transmission patterns after implementing random down-sampling to mitigate sampling bias. This approach limits representation to a maximum of ten genomes per country, providing a more balanced view of international transmission dynamics. (C-F) Phylogeographic analyses of four random subsets of the down-sampled data (subsets 55, 44, 33, and 22). Each tree is colored according to the inferred geographic origin of isolates, with branches representing transitions between countries. The country labels on the right side of panel C apply to all four subset analyses.





## **Figure S7 Geographic clustering of carbapenemase-carrying plasmids.** (A) Phylogenetic analysis of the *bla*_KPC-2_-carrying plasmid PT_3142. The tree shows clear geographic clustering with nodes colored according to country of origin. The Singapore isolates (blue nodes, *n* = 176) form a remarkably tight phylogenetic cluster highlighted by the red circle, despite being hosted by *K. pneumoniae* strains from five different clades. Numbers in brackets indicate the sample size from each country. (B) Phylogenetic analysis of the *bla*_OXA-48_-carrying plasmid PT_804. Nodes are colored according to continent of origin, with a strong predominance of European isolates (blue, *n* = 590) forming distinct clusters. The largest cluster (highlighted by the red circle) contains closely related plasmids from various European countries, clearly separated from Asian isolates. Numbers in brackets indicate the sample size from each continent.





**Figure S8 Distribution of virulence gene integrity across carbapenemase-producing *K. pneumoniae* isolates.** (A-H) Histogram of intact virulence genes across CC23-K1 isolates carrying different carbapenemase genes. The x-axis represents different carbapenemase types (NDM-1, NDM-5, OXA-48, KPC-2, and others), while the y-axis shows the percentage of isolates with intact virulence genes. (I) Histogram of virulence genes in CRKP clusters and singletons separated by carbapenemase types (x-axis). (J and K) Heatmaps of virulence gene prevalence across different carbapenemase-carrying *K. pneumoniae* lineages: (J) ST11 lineage and (K) other *K. pneumoniae* lineages excluding CC23/ST11. The color scale represents the proportion of isolates (0-1) containing intact virulence genes, with red indicating high prevalence and blue indicating low prevalence. Rows represent different carbapenemase combinations, while columns indicate specific virulence genes (*ybt*, *clb*, *iuc*, *iro*, *rmp*, and *rmpA2*).


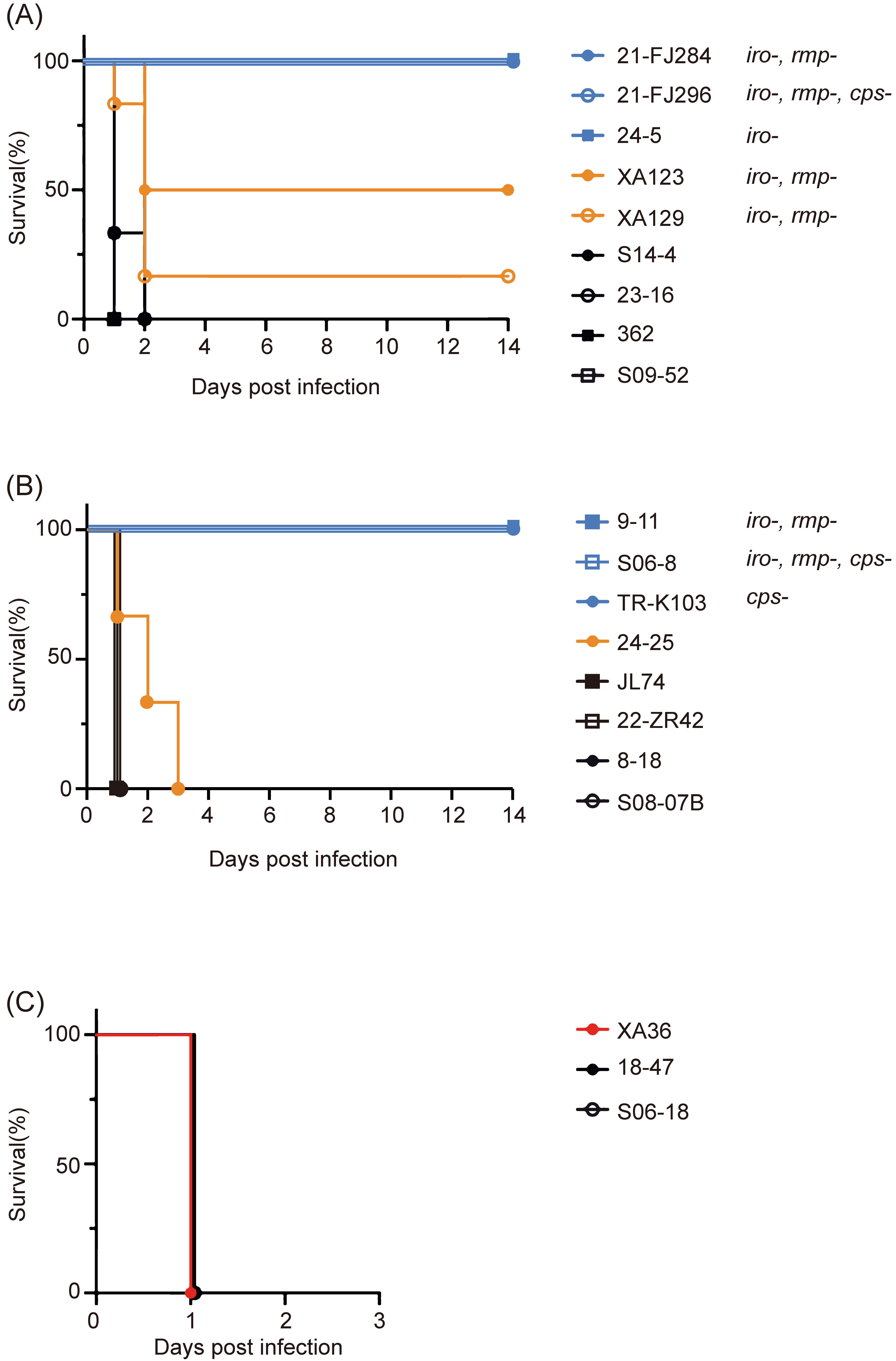


## **Figure S9 Assessment of *K. pneumoniae* strains virulence by intraperitoneal infection of mice.** (A-C) C57BL/6J mice were intraperitoneally infected with 5 × 10^5^ CFU of different *K. pneumoniae* strains. Each group was observed over a 14-day period. Survival rate was analyzed by Kaplan-Meier method with a log-rank test.





**Figure S10 Analysis of highly mutated regions and metabolic pathway enrichment in *K. pneumoniae* lineages.** (A) Volcano plot comparing highly mutated regions in ST11 *K. pneumoniae*. The x-axis represents log2(Fold Change) and the y-axis shows -log10(*p*-value). Blue dots indicate genes, while orange dots represent intergenic regions. The plot demonstrates that ST11 has a distinct pattern of highly mutated regions compared to CC23-K1. (B) Pathway enrichment analysis of highly mutated regions (HMRs) in CC23-K1. The y-axis shows GeneRatio, while the dot size indicates the count of genes involved in each pathway. The color scale represents adjusted *p*-values, with red indicating more significant enrichment. (C) The specific genes and metabolic pathways involved in the enriched pathways. The left section details the phosphotransferase system and the right section depicts the inositol phosphate metabolism pathway. (D) The specific genes and metabolic pathways involved in the enriched pathways in KPC/NDM-encoding isolates. The left section details the oxidative phosphorylation system and the right section depicts the menaquinone synthesis system.
